# Insula response to unpredictable and predictable aversiveness in individuals with panic disorder and comorbid depression

**DOI:** 10.1186/2045-5380-4-9

**Published:** 2014-10-07

**Authors:** Stephanie M Gorka, Brady D Nelson, K Luan Phan, Stewart A Shankman

**Affiliations:** 1 Department of Psychology, University of Illinois-Chicago, 1007 West Harrison St. (M/C 285), Chicago, IL 60607, USA; 2 Department of Psychology, Stony Brook University, Stony Brook, NY 11794, USA; 3 Department of Psychiatry, University of Illinois-Chicago, 1747 West Roosevelt Road, Chicago, IL 60608, USA; 4 Mental Health Service Line, Jesse Brown VA Medical Center, 820 S. Damen Avenue, Chicago, IL 60612, USA

**Keywords:** Insula, Unpredictable aversiveness, Panic disorder, Depression

## Abstract

**Background:**

Prior studies suggest that hyperactive insula responding to unpredictable aversiveness is a core feature of anxiety disorders. However, no study to date has investigated the neural correlates of unpredictable aversiveness in those with panic disorder (PD) with comorbid major depressive disorder (MDD). The aim of the current study was to examine group differences in neural responses to unpredictable and predictable aversiveness in 41 adults with either 1) current PD with comorbid MDD (PD-MDD), 2) current MDD with no lifetime diagnosis of an anxiety disorder (MDD-only), or 3) no lifetime diagnosis of psychopathology. All participants completed a functional magnetic resonance imaging (fMRI) scan while viewing temporally predictable or unpredictable negative or neutral images.

**Findings:**

The results indicated that individuals with PD-MDD exhibited greater bilateral insula activation to unpredictable aversiveness compared with controls and individuals with MDD-only (who did not differ). There were no group differences in insula activation to predictable aversiveness.

**Conclusions:**

These findings add to a growing literature highlighting the role of the insula in the pathophysiology of anxiety disorders.

## Findings

### Introduction

Heightened anticipatory responding to uncertain negative events is a hallmark feature of clinical anxiety [1]. Neuroimaging research indicates that the insula plays a major role in responding to uncertainty [2-4]. The insula is involved in interoceptive awareness and anticipatory emotional responses for future-oriented events [5]. It also plays a role in the generation of how future events will feel by guiding predictions about the salience of upcoming aversiveness [6].

Individuals with anxiety disorders (e.g., post-traumatic stress disorder, generalized anxiety disorder, social anxiety disorder) exhibit hyperactive anterior and middle insula activation during the anticipation of unpredictable negative events [7-9]. Individuals at risk for anxiety disorders also display this pattern of results [10,11]. However, no study to our knowledge has examined the neural correlates of unpredictable aversiveness in those with panic disorder (PD)—an anxiety disorder characterized by heightened anticipatory anxiety in response to unpredictable panic attacks [12]. One would strongly speculate that this population exhibits abnormal insula reactivity to unpredictable aversiveness; however, empirical data is needed to corroborate this hypothesis.

An additional, more serious gap in the literature is that no study has examined the impact of comorbid major depressive disorder (MDD) on the neural correlates of unpredictable aversiveness in those with *any* anxiety disorders. This is a critical omission given that depression and anxiety share many neurobiological features [13] and the broader psychophysiological literature on the impact of comorbid depression on aversive responding in anxiety disorders is extremely mixed. It has been demonstrated that comorbid MDD has no impact [14], blunts aversive responding [15,16], and enhances aversive responding [17,18]. Given these findings, it is necessary that studies examine the role of comorbid PD and MDD, and MDD with no lifetime diagnosis of an anxiety disorder (MDD-only) on neural responses to aversiveness.

The aim of the current study was to examine neural responses to predictable and unpredictable aversiveness using functional magnetic resonance imaging (fMRI) in three groups: 1) current PD with comorbid MDD, 2) current MDD-only, and 3) no lifetime history of psychopathology. We used a passive picture-viewing task, previously shown to probe insula responses [3], during which participants anticipated viewing temporally predictable and unpredictable negative or neutral images. We hypothesized that individuals with PD-MDD would exhibit greater bilateral insula activation to unpredictable, but not predictable, aversiveness compared with MDD-only and control participants (who would not differ from each other).

### Methods

#### *Participants*

The study included 41 adults with either 1) current PD with comorbid MDD (*n* = 13), 2) current MDD with no lifetime diagnosis of an anxiety disorder (*n* = 9), or 3) no lifetime history of psychopathology (*n* = 19). Participants were recruited from a larger study on emotional processes [14]. Clinical diagnoses were made using the Structured Clinical Interview for *DSM-IV*[19]. Participants in the comorbid group, but not the MDD-only group, were allowed to have additional lifetime anxiety disorders (Table 1). All participants provided written informed consent after review of the protocol and procedures were approved by the University of Illinois at Chicago Institutional Review Board.

**Table 1:** Demographics and clinical characteristics

	**Controls (*n *** **= 19)**	**MDD-only (*n *** **= 9)**	**PD-MDD (*n *** **= 13)**
Demographic variables			
Age (years, *SD*)	29.6 (12.8) a	25.4 (7.7) a	39.1 (11.9) b
Sex (% female)	68.4 a	66.7 a	76.9 a
Race (% Caucasian)	52.6 a	22.2 a	41.7 a
Education			
Graduated high school or GED (%)	5.3	0.0	0.0
Part college or graduated 2-year college (%)	21.1	55.6	50.0
Graduated 4-year college (%)	47.4	33.3	33.3
Part or completed graduate/professional school (%)	26.2	11.1	16.6
Current comorbid diagnoses			
Social phobia (%)	-	-	23.1
Specific phobia (%)	-	-	7.7
Post-traumatic stress disorder (%)	-	-	7.7 a
Generalized anxiety disorder (%)	-	-	0.0 a
Obsessive compulsive disorder	-	-	7.7 a
Current psychiatric medications (%)	0.0 a	11.1 a	30.8 b

*Note.* Means or percentages with different letters across rows were significantly different in pairwise comparisons (*p* < 0.05, chi-square test for categorical variables and Tukey’s honestly significant difference test for continuous variables). *Controls* individuals with no history of psychopathology, *MDD-only* individuals with current major depressive disorder and no history of anxiety disorders, *PD-MDD* individuals with current comorbid panic disorder and major depressive disorder, *SD* standard deviation.

#### *Procedure and aversiveness task*

The aversiveness task has been described in detail elsewhere [3]. The participants viewed a series of count-ups (CU; e.g., 1-2-3) that ended with the presentation of a negative or neutral image selected from the International Affective Picture System (IAPS) [20]. The task included two within-subjects factors—timing (predictable (P) vs. unpredictable (U)) and valence (negative (Neg) vs. neutral (Neut)). For each trial, text initially appeared at the bottom of the screen for 2 s indicating the timing and valence (i.e., P-Neut, P-Neg, U-Neut, or U-Neg). Next, the CU was presented for 4 to 11 s. At the end of the CU, the image appeared for 1.5 s. In the P condition, the participants were told when the CU would end and the valence of the image that would appear (e.g., “Neutral image at 6”). In the U condition, the participants knew the valence but did not know when the image would appear (e.g., “Unpleasant image can appear at anytime”). For each condition, trials were presented during 42-s blocks during which the CU was presented four times. Each condition block was presented four times, counterbalanced across two runs. In between blocks, a fixation cross was presented for 10 s.

#### *fMRI data acquisition*

Functional gradient-echo echo-planar images were acquired during the task (2 s TR, 25 ms TE, 82° flip, 64 × 64 matrix, 200-mm field of view (FOV), 3-mm slice thickness, 0-mm gap, with 40 axial slices). A high-resolution, T1-weighted anatomical scan was also acquired in the same axial orientation (25° flip, 512 × 512 matrix, 220-mm FOV, 1.5-mm slice thickness, 120 axial slices).

#### *fMRI data analyses*

All data met criteria for high quality and scan stability with minimum motion correction (i.e., <3-mm displacement in any one direction). Functional data were analyzed using Statistical Parametric Mapping software (SPM8, Wellcome Department of Imaging Neuro-Science, London, UK). The images were spatially realigned, warped to standardized Montreal Neurological Institute (MNI) space using each participant’s T1 image, resampled to 2-mm^3^ voxels, and smoothed with an 8-mm^3^ kernel. The general linear model was applied to the time series, convolved with the canonical hemodynamic response function and with a 128-s high-pass filter. Effects were estimated at each voxel and for each subject.

CUs and presentation of IAPS images during U-Neg, U-Neut, P-Neg, and P-Neut conditions were modeled separately at the first level. Individual statistical parametric maps for CUs only were entered into a second-level 2 (valence: Neg vs. Neut) × 2 (predictability: P vs. U) × 3 (group: control vs. MDD-only vs. PD-MDD) analysis of variance (ANOVA). Given our hypotheses about the role of predictability, if we did not find any significant three-way interactions, we ran separate 2 (valence) × 3 (group) ANOVAs for P and U conditions. Age was included as a covariate in all analyses. Because of our *a priori* hypothesis about the insula, we created an anatomically derived partial brain mask of the entire bilateral insula (5,731 voxels) and applied a cluster-based significance thresholding to adjust for multiple comparisons. Based on simulations (10,000 iterations) performed with AlphaSim (http://afni.nimh.nih.gov/pub/dist/doc/manual/AlphaSim.pdf), a familywise error correction at *α* < 0.05 is achieved with a voxel threshold of *p* < 0.005 and a cluster size of at least 61 contiguous voxels. We extracted BOLD signal responses (arbitrary units) from 5-mm (radius) spheres surrounding significant peak activations to conduct *post hoc* comparisons.

### Results

#### *Behavioral results*

Negative images were rated as more unpleasant (*F*(1, 38) = 98.25, *p* < 0.01) and arousing (*F*(1, 38) = 18.91, *p* < 0.01) relative to neutral images. Valence and arousal ratings did not differ as a function of predictability (all *p*s > 0.05). PD-MDD participants rated neutral images as more arousing relative to MDD-only (*F*(1, 20) = 4.34, *p* < 0.05) and control (*F*(1, 30) = 6.63, *p* < 0.05) participants (who did not differ).

#### *Imaging results*

**Figure 1:**
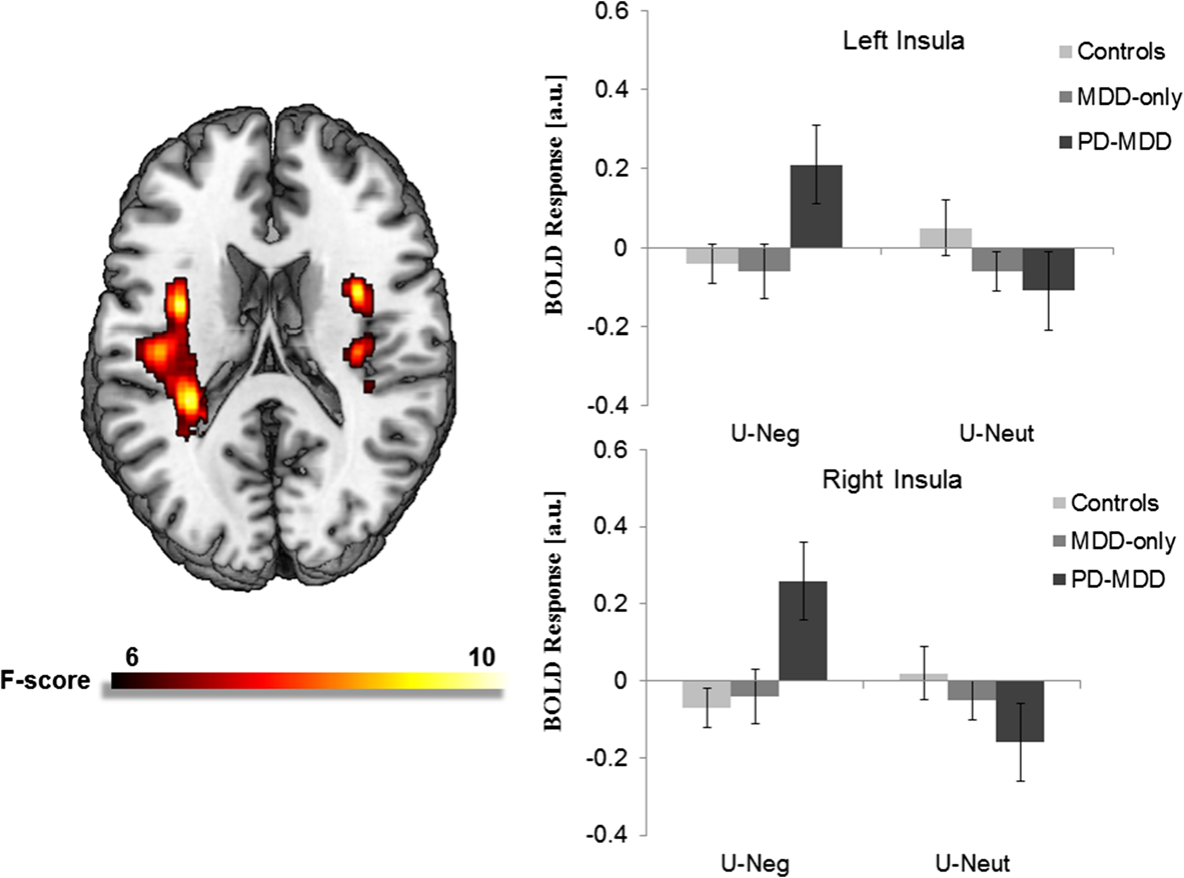
**Voxelwise statistical *F*-map on a canonical brain displaying significant valence × group interactions in neural responses to unpredictable conditions.** Color scale reflects *F*-value. Bar graph illustrating extracted parameter estimates from the left and right insula cortices during anticipation of unpredictable negative images and unpredictable neutral images. *Controls* no history of psychopathology, *MDD-only* current diagnosis of major depressive disorder and no lifetime history of an anxiety disorder, *PD-MDD* current diagnoses of major depressive disorder and panic disorder.

There were no significant valence × predictability × group interactions. However, there was a significant valence × group interaction for bilateral middle insula activation during the U conditions (right MNI peak [34, -20, 20], *Z* = 3.48, *p* < 0.05, corrected; left MNI peak [-36, -2, 18], *Z* = 3.78, *p* < 0.05, corrected; see Figure 1). Specifically, the groups differed on bilateral middle insula activation during U-Neg (right *F*(2, 40) = 4.72, *p* < 0.05; left *F*(2, 40) = 3.81, *p* < 0.05), but not during U-Neut (*p*s > 0.05). During U-Neg, the PD-MDD group exhibited greater bilateral insula activation compared with controls (right *t*(30) = 2.71, *p* < 0.05; left *t*(30) = 2.36, *p* < 0.05) and MDD-only subjects (right *t*(20) = 2.11, *p* < 0.05; left *t*(20) = 2.20, *p* < 0.05). The control and MDD-only participants did not differ from each other. Moreover, there were no significant valence × group interactions during the P conditions. All whole-brain results are presented in Table 2.

**Table 2:** Whole-brain results for the valence-by-group analysis of variance during the unpredictable and predictable conditions

**Model**	**Region**	**MNI coordinates**			**Voxels**	***Z*-score**
		***X***	***Y***	***Z***		
Group by valence interaction						
U conditions	R medial frontal gyrus	14	-16	58	157	3.80
	L insula	-36	-2	18	1,162	3.78
	R insula	34	-20	20	315	3.48
P conditions	L medial frontal gyrus	-16	-4	54	136	3.72
	R inferior frontal lobe	2	0	-18	456	3.57
	L parietal lobe	-28	-42	42	292	3.53
	L inferior temporal lobe	-46	-54	-10	413	3.44
	L posterior cingulate cortex	-8	-40	34	198	3.05
Main effect of group						
U conditions	L cerebellum	-20	-84	-32	118	3.15
P conditions	L lingual gyrus	-18	-80	-4	223	4.05
Main effect of valence						
U conditions	R middle frontal gyrus	54	18	40	6,501	5.48
	R inferior frontal gyrus	36	22	-18	23,489	5.13
	L middle frontal gyrus	-42	6	58	5,435	4.48
	R medial frontal gyrus	6	44	42	669	4.31
	R cuneus	4	-98	6	221	3.27
P conditions	R frontal lobe	22	-4	28	138	3.32
	R cerebellum	24	-50	-38	67	3.16

*Note*. Reporting of all clusters exhibiting significance at *p* < 0.05 (corrected). *L* left, *R* right, *MNI* Montreal Neurologic Institute, *U conditions* anticipation of unpredictable negative and neutral images, *P conditions* anticipation of predictable negative and neutral images.

### Discussion

Consistent with our hypotheses, individuals with PD-MDD exhibited greater bilateral insula activation to unpredictable aversiveness compared with controls and individuals with MDD-only (who did not differ from each other). There were no group differences in insula activation to predictable aversiveness. Although the group by valence by predictability interaction was not significant, this pattern of results suggests that the association between PD-MDD and hyperactive insula responding may be more robust during anticipation of unpredictable relative to predictable aversiveness.

Given that individuals with PD-MDD, but not individuals with MDD-only, exhibited hyperactive insula responding, PD may be associated with enhanced insula reactivity to uncertain aversiveness similar to other anxiety disorders. This is noteworthy given that PD is characterized by chronic heightened anticipatory anxiety between panic attacks [12]. Moreover, after experiencing an initial panic attack, individuals develop PD via a process in which anticipatory anxiety regarding the temporal uncertainty of the next panic attack increases the likelihood of additional attacks [21]. A positive feedback loop between anticipatory anxiety and panic attacks is thought to precipitate the onset of PD. In light of the current findings, it is possible that heightened insula reactivity maintains chronic anticipatory anxiety and is a brain-based mechanism underlying the transition from initial panic attack to PD. Although it is also possible that heightened insula reactivity is a consequence of PD and/or a concomitant of the disorder, individuals at risk for anxiety disorders have similarly demonstrated this effect, suggesting that heightened insula reactivity to unpredictable aversiveness may indeed be a biological risk factor [11]. Future research is therefore needed to further elucidate the role of insula reactivity in PD.

These results also fit with current theory on the functions of the insula [5,22]. In response to uncertain aversiveness, individuals with PD-MDD exhibited hyperactivation of the middle insula, which is a region responsible for integrating environmental and interoceptive information to ultimately represent moments in time and make inferences about how future events will feel. Among individuals with PD, heightened middle insula reactivity may reflect a tendency to overestimate the affective consequences of impending aversiveness, resulting in exaggerated anticipatory anxiety [1]. Importantly, this process is consistent with the clinical picture of PD as these individuals tend to overestimate the harm of panic attacks, which produces anticipatory anxiety between attacks [23]. Heightened insula reactivity to uncertain aversiveness may therefore contribute to the onset of PD (noted above) and the maintenance of PD.

Although these findings address important gaps in the literature, there are several limitations. First, the current sample size was small, which reduced statistical power, and the results should therefore be considered preliminary. Second, approximately one third of the comorbid subjects were currently taking psychiatric mediations, and it is possible that this impacted their neural responding. Notably, when individuals currently taking medications were excluded from the current study, the pattern of results was entirely the same. Third, future research is needed to determine whether the current findings are due to PD or PD-MDD.

The current study has several important implications. Most notably, the results indicate that individuals with PD-MDD exhibit heightened insula reactivity to unpredictable aversiveness compared with healthy controls. This adds to a growing literature noting that hyperactive insula responding to unpredictable aversiveness may contribute to the pathophysiology of anxiety disorders.

## Abbreviations

PD: panic disorder; MDD: major depressive disorder; PD-MDD: comorbid panic disorder and major depressive disorder; CU: count-ups; P: predictable; U: unpredictable; Neg: negative; Neut: neutral; MNI: Montreal Neurological Institute.

## Competing interests

The authors declare that they have no competing interests.

## Authors’ contributions

SAS designed the study and wrote the protocol. SMG wrote the first draft of the manuscript and conducted the analyses. BDN conducted the literature search and assisted in the editing of the manuscript. KLP helped with the interpretation of the results and made important contributions to the editing of the manuscript. All authors contributed to and have approved the final manuscript.
